# Identification of immune and metabolic predictors of severe hand-foot-mouth disease

**DOI:** 10.1371/journal.pone.0216993

**Published:** 2019-05-23

**Authors:** Luwei Qin, Dejian Dang, Xinhong Wang, Rongguang Zhang, Huifen Feng, Jingchao Ren, Shuaiyin Chen, Guangyuan Zhou, Ping Huang, Bin Wang, Yuanlin Xi, Weidong Wu, Yuefei Jin, Guangcai Duan

**Affiliations:** 1 School of Public Health, Xinxiang Medical University, Xinxiang, China; 2 Department of Epidemiology, College of Public Health, Zhengzhou University, Zhengzhou, China; 3 Department of Infectious Disease, The Children's Hospital of Zhengzhou City, Zhengzhou, China; 4 Department of Gastroenterology, The Fifth Affiliated Hospital of Zhengzhou University, Zhengzhou, China; The University of Hong Kong, CHINA

## Abstract

Hand, foot and mouth disease (HFMD) is an infectious disease that affects mostly children. The children with HFMD also have other immune and metabolic disorders. However, the association of these disorders with the severity of HFMD has not yet been determined. In this study, we used a case-control study design to examine the correlation of immune and metabolic disorders with HFMD development in children. 406 mild and severe patients were recruited and divided into different subgroups based on the number of days from the initial onset time to hospitalization (1, 2, 3, 4, and ≥5 days). Logistic regression model was used to define the predictors of severe HFMD. We found that the patients from rural area (OR = 1.76, 95% CI [1.19~2.63], *P* = 0.005) or with body temperature of >39°C (OR = 2.14, 95% CI [1.12~4.12], *P* = 0.022) exhibited higher risk for severe symptoms. In addition, the risk increased with the rise of body temperature by using a Chis-quare trend test (*P* = 0.01). We also found that a decreased number of eosinophils was an predictor of severe HFMD at 1, 2, 3,and 4 days post infection (dpi). Decreased levels of Na^+^, Cl^-^, and creatine kinase were also predictors at 1 and ≥5 dpi. On the other hand, elevated level of globulin was a predictor for severe HFMD at 4 dpi and ≥5 dpi, and the increased number of neutrophils or increased level of alkaline phosphatase posed risk for severe HFMD at 3 and ≥5 dpi. Our results suggested that rural living, hyperpyrexia, changes in the immune system that include the numbers of eosinophils and neutrophils and the levels of IgG and globulin, and metabolic alterations, such as the levels of alkaline phosphatase, Na^+^, Cl^-^, and creatine kinase in peripheral blood are predictors of severe HFMD.

## Introduction

Hand, foot and mouth disease (HFMD) is a common pediatric infectious disease caused by members of *Picornaviridae* family such as enterovirus 71 (EV71) and coxsackie A16 (CA16) that usually affect infants and young children worldwide [1, 2].Due to lack of effective therapies or vaccines, millions of children in the world still suffer from HFMD every year [2, 3]. Generally, the majority of HFMD cases are usually mild and self-limiting, but a few cases can develop into devastating clinical outcomes with neural infection, and even fatal cardiopulmonary failure [4–6]. It has been reported that the mortality of severe HFMD was as high as 3.0% from 2008 to 2012 in China mainland [7]. Although the inactivated EV71 vaccine has been widely used to vaccinate infants and young children in several provinces of mainland China and has shown to be effective at protecting them from EV71-associated severe HFMD, the number of severe cases is still large, posing a threat to infants and young children in rural areas of Asia-Pacific region [4–6, 8].

Risk factors can influence the probability that a specific disease develops. Since HFMD emerged, many studies have documented that demographic characteristics, clinical symptoms and signs, rural living, long interval from the onset of illness to being hospitalized, serious conditions when patients are admitted to hospital, clinical and laboratory indicators, and other factors are risk factors of severe HFMD [6, 7, 9]. Immune and metabolic changes have been detected in HFMD patients [10, 11]. However, the association of these changes with HFMD severity has not been demonstrated. Here, we analyzed 406 mild and severe cases that were divided into different groups based on the time of hospitalization and initial onset (1, 2, 3, 4, and ≥5 days post infection). We analyzed the numbers of immune cells, and the levels of globulin, immunoglobulin G (IgG), systematic inflammatory markers, and metabolic parametersin peripheral blood from these patients by a case-control study. Logistic regression was used to study the predictors of severe HFMD. Understanding what immune and metabolic changes contribute to the risk of severe HFMD will help to prevent the development of this severe disease in children and to better inform public health and improve clinical practice.

## Methods

### Ethical statement

This study was approved by the Life Sciences and Ethics Committee of Zhengzhou University (Approval NO. 2014–0911) and the Ethics Committee of the Children’s Hospital of Zhengzhou (Approval NO. 2013–0816). All procedures were conducted in accordance with the Declaration of Helsinki. Written informed consent was obtained from parents or guardians of all the participants included in the study.

### Subjects

A total of 406 subjects including 178 mild cases and 228 severe cases was enrolled in this study. These patients were admitted in the Children's Hospital of Zhengzhou City from January 2015 to December 2016. HFMD cases were diagnosed following “hand, foot and mouth disease treatment guidelines” (Chinese Ministry of Public Health, revised in 2010). The cases with encephalitis, acute flaccid paralysis, myocarditis, and pulmonary edema were classified as severe cases [11], and the cases without above symptoms were classified as mild cases. Additionally, the patients with congenital heart disease, pneumonia, and autoimmune disease were excluded from this study.

### Data collection and grouping

Demographic characteristics and clinical symptoms were collected by the physicians through retrospective medical records and face-to-face interviews with children’s parents during the admission. The clinical information of HFMD cases was collected after onset of illness, but before diagnosis of severe HFMD or fatality. Laboratory indicators were collected from general blood tests for clinical diagnosis. The authors had access to information that could identify individual participants during or after data collection. All subjects were divided into different subsets of 1, 2, 3, 4, and ≥5 days post infection (dpi) based on the hospitalization and initial onset time.

### Statistical analysis

Data were double-entered and validated using EpiData version 3.1 (the EpiData Association, Denmark) and analyzed withR version 3.2.3 (R Development Core Team, 2015, R Foundation for Statistical Computing, Vienna, Austria). The mild and severe cases were divided into different subgroups of 1, 2, 3, 4, and ≥5 days post infection (dpi) based on our previous work [11]. Differences between groups or subgroups were tested by Student’s *t* test or Kruskal-Wallis test according to the distribution of data. Chis-quare test or Fisher’s exact test was used to test the differences in the proportions of categorical variables. Significant differences were brought into a multivariable regression model built in backward stepwise fashion. A *p*-value <0.05 was considered of significant difference. The packages used in R were ggplot2 [12] and forestplot [13] graphic system.

## Results

### Association of rural living and hyperpyrexia with the severity of HFMD

There was no significant difference in sex ratio (male ratio, mild: 114/178; severe: 137/228, χ^2^ = 0.663, *P* = 0.415) and age (mild: 21.08±10.70; severe: 22.04±11.40) between mild and severe groups. The geographical origin and body temperature of mild and severe cases are listed in **Table 1**. Although there was an extremely high rate of eruption (a manifestation of skin lesion) (99.01%) in the subjects, our results indicated that it was not associated with the occurrence of severe symptoms. Rural patients exhibited higher risk for severe symptoms (OR = 1.76,95%CI [1.19~2.63], *P* = 0.005). In addition, >39°C body temperature was also a predictor of severe symptoms (OR = 2.14,95%CI [1.12~4.12], *P* = 0.022), and the risk was increased with the rise of body temperature by using a Chisquare trend test (*P* = 0.01).

**Table 1:** The geographical origin and body temperature of mild and severe cases.

Variable	Mild(n = 178)	Severe(n = 228)	Z	p-value	OR(95%CI)
Inhabitancy(rural) n,% Body temperature	82(46.07)	137(60.09)	2.80	0.005	1.76(1.19~2.63)
<37.5°C	28	24	-	-	1
37.5~38.0°C	12	11	0.13	0.894	1.07(0.40~2.87)
38.1~39.0°C	90	105	0.99	0.325	1.36(0.74~2.53)
>39.0°C	48	88	2.30	0.022	2.14(1.12~4.12)
Eruption n,%	177(99.44)	225(98.68)	0.74	0.459	0.42(0.02~3.34)

### Immune and metabolic changes in the peripheral blood of mild and severe patients

To evaluate the association of immune and metabolic changes with the severity of HFMD, we analyzed the data of blood tests from clinical diagnosis. To detect changes in the immune system, we determined the numbers of blood cells, including WBC, RBC, platelet, neutrophils, lymphocytes, monocytes, eosinophils, and basophils, and the concentrations of IgM, IgA, IgG, C3, and C4 in the peripheral blood from mild and severe cases. For metabolic changes, we assayed the levels of CRP, neuron-specific enolase, bilirubin (total, direct, and indirect bilirubin), ALT, AST, ALP, γ-GT, total protein, ALB, GLO, LDH, HBDH, CK, CK-MB, blood urine nitrogen, and creatinine as listed in **Table 2**. In comparison with mild cases, the numbers of PLT, neutrophils and the levels of ALP, total protein, globulin, and IgG were significantly increased in severe cases, while the numbers of monocytes, eosinophils, basophils, and the levels of C-reactive protein (CRP), CK, CK-MB, Na^+^, and Cl^-^ were reduced. Thus, our data suggest that these immune and metabolic changes can serve as potential predictors of severe HFMD.

**Table 2:** Laboratory findings in peripheral blood from mild and severe cases.

Variable	Mild(n = 178)	Severe(n = 228)	*p*-value
WBC(10^9^/L)	9.59 (7.25,11.97)	9.30 (7.56,11.79)	0.940
RBC(10^12^/L)	4.52(4.33,4.78)	4.53(4.28,4.74)	0.740
PLT(10^9^/L)	309.50(250,369)	331.50(279.25,389.50)	0.002
NEUT(10^9^/L)	4.30(2.56,6.79)	5.12(3.36,6.93)	0.010
LYMPH(10^9^/L)	3.68(2.70,5.52)	3.50 (2.38,4.96)	0.064
MONO(10^9^/L)	0.53(0.32,0.78)	0.46(0.26,0.68)	0.036
EO(10^9^/L)	0.02(0.00,0.12)	0.00(0.00,0.01)	<0.001
BASO(10^9^/L)	0.02(0.01,0.03)	0.01(0.01,0.02)	0.020
CRP(mg/L)	5.60 (1.28,15.44)	2.69(0.80,13.11)	0.002
NSE(ng/mL)	29.83(25.25,36.29)	28.04(24.12,35.20)	0.345
TBIL(μmol/L)	7.30(6.20,9.40)	7.55(6.00,9.80)	0.810
DBIL(μmol/L)	3.10(2.13,3.90)	3.00(2.00,3.80)	0.228
IBIL(μmol/L)	4.70(3.53,5.80)	4.90(3.50,6.40)	0.479
ALT(U/L)	14.50(11.00,19.00)	15.00(11.00,20.00)	0.719
AST(U/L)	33.00(28.00,38.00)	31.00(27.00,37.00)	0.197
ALP(U/L)	180.50(148.25,215.00)	201.00(166.50,232.00)	0.001
γ-GT(U/L)	14.00(11.00,16.00)	14.00(10.75,16.25)	0.687
TP(g/L)	68.50(65.53,72.58)	72.05(68.18,76.45)	<0.001
ALB(g/L)	46.20(44.20,47.90)	46.25(43.58,48.70)	0.984
GLO(g/L)	22.90(20.90,25.33)	25.00(22.70,28.83)	<0.001
LDH(U/L)	292.00(255.00,339.00)	275.50(241.70,346.00)	0.133
HBDH(U/L)	251.00(227.50,278.50)	246.00(224.00,272.00)	0.381
CK(U/L)	84.00(56.00,116.50)	66.50(47.00,100.00)	0.001
CK-MB(U/L)	20.00(15.00,25.00)	18.00(13.00,23.00)	0.037
BUN(mmol/L)	3.35(2.73,4.18)	3.60(2.88,4.23)	0.206
CREA(μmol/L)	24.00(21.00,28.00)	25.00(21.00,28.00)	0.677
UA(μmol/L)	278.00(231.00,338.00)	276.50(226.75,336.00)	0.903
K(mmol/L)	4.21(4.02,4.47)	4.23(3.94,4.47)	0.439
Na(mmol/L)	138.00(137.00,139.00)	137.00(135.00,138.00)	<0.001
Cl(mmol/L)	102.00(100.00,103.00)	101.00(99.00,102.00)	<0.001
Ig M(g/L)	0.92(0.70,1.17)	0.97(0.74,1.24)	0.077
Ig A(g/L)	0.34(0.23,0.59)	0.36(0.22,0.59)	0.890
Ig G(g/L)	6.67(5.62,7.95)	7.39(5.85,9.40)	0.006
C3(g/L)	1.10(0.96,1.26)	1.14(1.00,1.27)	0.273
C4(g/L)	0.26(0.21,0.32)	0.26(0.21,0.33)	0.750

**Abbreviations:** WBC: white blood cell, RBC: red blood cells, PLT: platelet, NEUT: neutrophils, LYMPH: lymphocytes, MONO: monocytes, EO: eosinophils, BASO: basophils, CRP: C-reactive protein, NSE: neuron-specific enolase, TBIL: total bilirubin, DBIL: direct bilirubin, IBIL: indirect bilirubin, ALT: alanine aminotransferase, AST: aspartate aminotransferase, ALP: alkaline phosphatase, γ-GT: glutamyl transpeptidase, TP: total protein, ALB: albumin, GLO: globulin, LDH: lactate dehydrogenase, HBDH: alpha-hydroxybutyrate dehydrogenase, CK: creatine kinase, CK-MB: creatine kinase isoenzyme, BUN: blood urine nitrogen, CREA: creatinine, UA: uric acid, IgM: Immunoglobulin M, IgA: Immunoglobulin A, IgG: Immunoglobulin G, C3: Complement 3, C4: Complement 4.

### Association of immune alterations with the severity of HFMD

**Fig 1:**
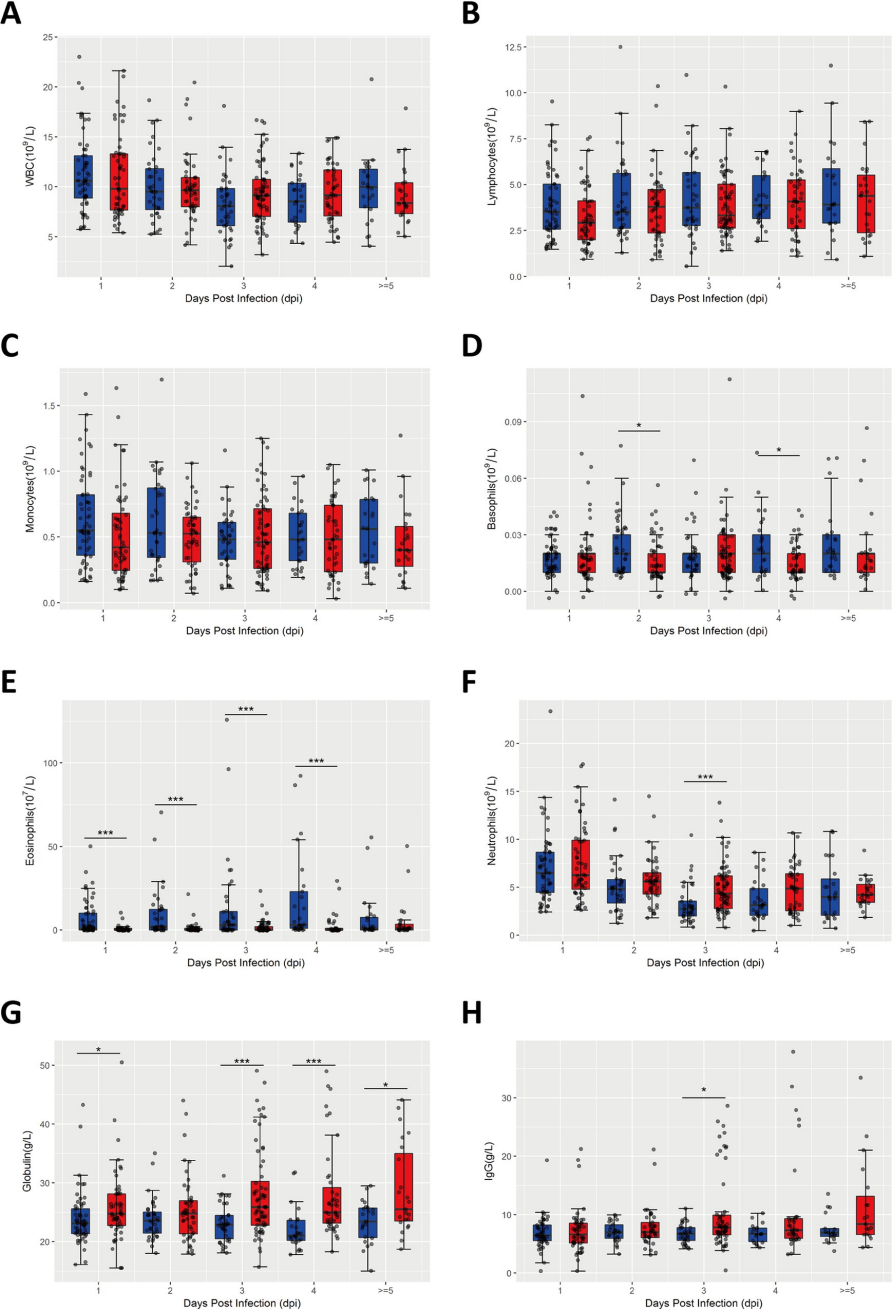
Association of immune changes with the severity of HFMD. The numbers of WBC (A), lymphocytes (B), monocytes (C), basophils (D), eosinophils (E), neutrophils (F), globulin (G), IgG (H) in the peripheral blood from mild (n = 178) and severe cases (n = 228) were analyzed based on different onset time points defined by retrospective medical records and face-to-face interviews with children’s parents during hospitalization. Data are expressed as means±SEM. *P<0.05, vs mild cases, ***P<0.05, vs mild cases.

Immune dysfunction is a main consequence of infectious diseases [14]. To study whether immune-related indicators had any association with the severity of HFMD, we analyzed the numbers of WBC, neutrophils, lymphocytes, monocytes, basophils, eosinophils in the peripheral blood from mild and severe cases. As shown in **Fig 1**, there was no change in the numbers of WBC (**Fig 1A**), lymphocytes (**Fig 1B**) and monocytes (**Fig 1C**) between mild and severe cases at different time points of onset. Compared to mild cases, the numbers of basophils (at 2, 4 dpi, **Fig 1D**) and eosinophils (at 1, 2, 3, 4 dpi, **Fig 1E**) in the peripheral blood of severe cases were all significantly reduced, while the number of neutrophils (**Fig 1F**) was significantly increased at 3 dpi. In addition, the levels of globulin (at 1, 3, 4, ≥5 dpi, **Fig 1G**) and IgG (at 3 dpi, **Fig 1H**) in peripheral blood of severe cases were all significantly elevated, a likely consequence of immune response to serious viral infection. Our results suggest that the inflammatory cells, such as basophils, eosinophils, and neutrophils, may play a significant role in HFMD development, and patients with severe HFMD likely have a stronger antiviral immune response.

### Association of metabolic changes with the severity of HFMD

**Fig 2:**
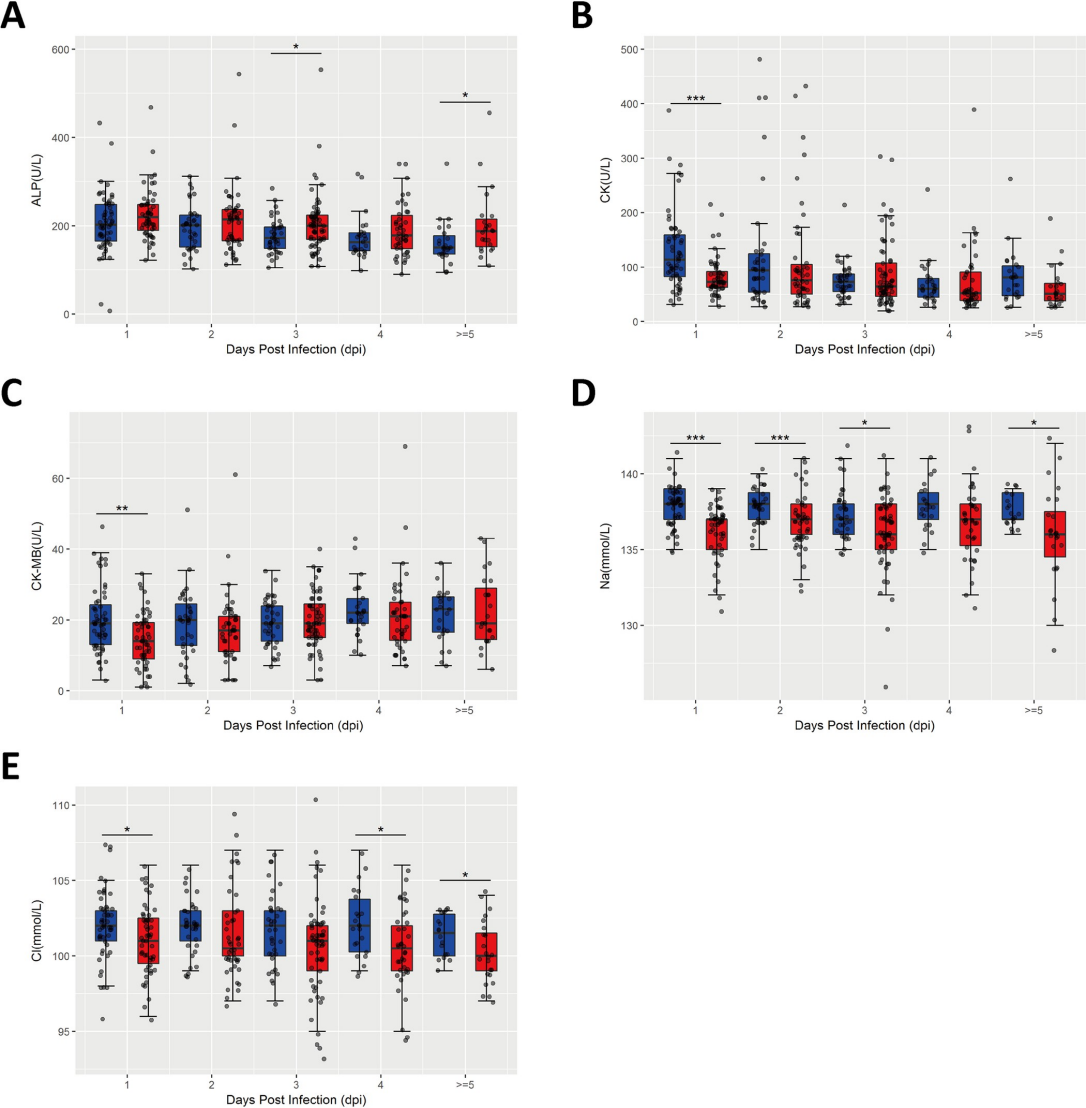
Association of metabolic changes with the severity of HFMD. The levels of alkaline phosphatase (ALP), creatine kinase (CK), creatine kinase isoenzyme (CK-MB), Na^+^, and Cl^-^ in the peripheral blood from mild (n = 178) and severe cases (n = 228) were analyzed at different onset time points defined by retrospective medical records and face-to-face interviews with children’s parents during the hospitalization time. Data are expressed as means ±SEM. *P<0.05, vs mild cases, **P<0.05, vs mild cases, ***P<0.05, vs mild cases.

To explore the kenetics of metabolic changes during progression of HFMD, we also analyzed the levels of alkaline phosphatase (ALP), creatine kinase (CK), creatine kinase isoenzyme(CK-MB), Na^+^, and Cl^-^in the peripheral blood from mild and severe cases as described above. As shown in **Fig 2A**, in comparison with mild cases, the level of ALP in the peripheral blood of severe cases was significantly increased at 3 dpi, while the levels of CK (at 1 dpi, **Fig 2B**), CK-MB (at 1 dpi, **Fig 2C**), Na^+^ (at 1, 2, 3, ≥5 dpi, **Fig 2D**), Cl^-^(at 1, 4, ≥5 dpi, **Fig 2E**) in the peripheral blood of severe cases were significantly reduced. Our data indicate that liver injury and electrolyte disorder happen in severe HFMD.

### Association of inflammatory biomarkers with the severity of HFMD

**Fig 3:**
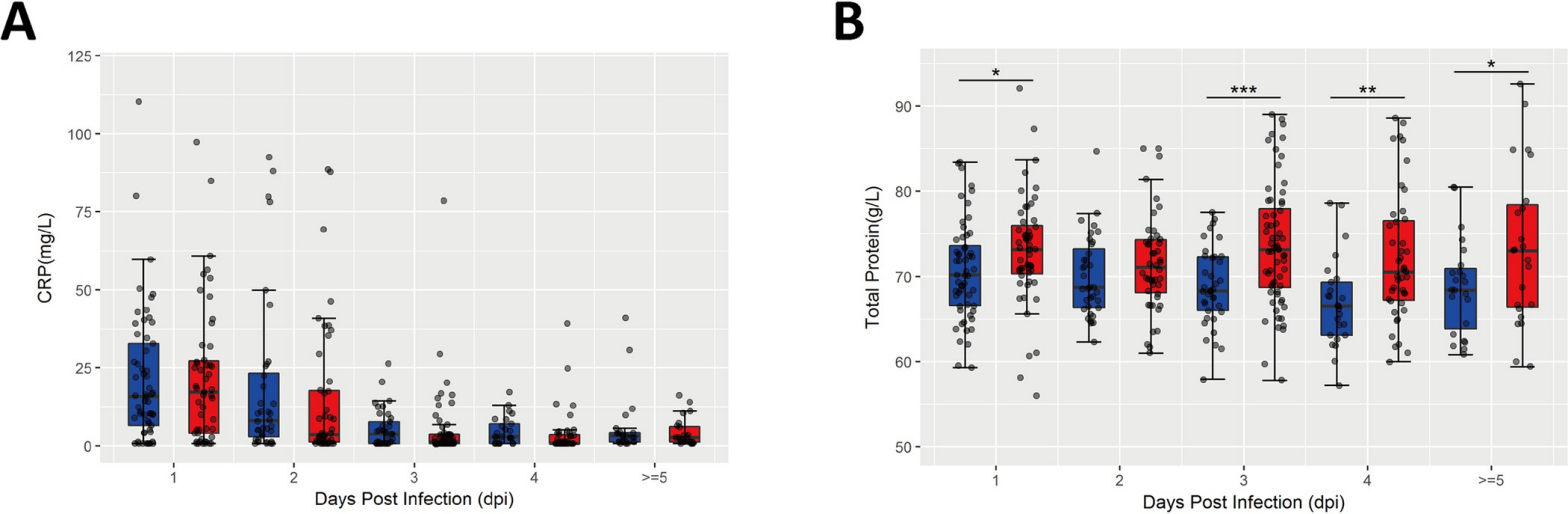
Association of inflammatory biomarkers with the severity of HFMD. C-reactive protein (CRP) (A). and total protein (B) were determined at different onset time points defined by retrospective medical records and face-to-face interviews with children’s parents during the hospitalization time. Data are expressed as means±SEM. *P<0.05, vs mild cases, **P<0.05, vs mild cases, ***P<0.05, vs mild cases.

C-reactive protein (CRP) and total protein are two important acute inflammatory biomarkers [15], and inflammation is well known to associate with HFMD development [11]. In the present study, we analyzed the levels of CRP and total protein in the peripheral blood from mild and severe cases based on different onset time points. As shown in **Fig 3A**, there was no significant difference in the level of CRP between mild and severe cases, while the level of total protein (**Fig 3B**) in severe cases was significantly elevated, compared to mild cases. These data indicate that severe cases exhibit more serious acute phase response.

### Multivariable logistic regression analysis of predictors of severe HFMD

**Fig 4:**
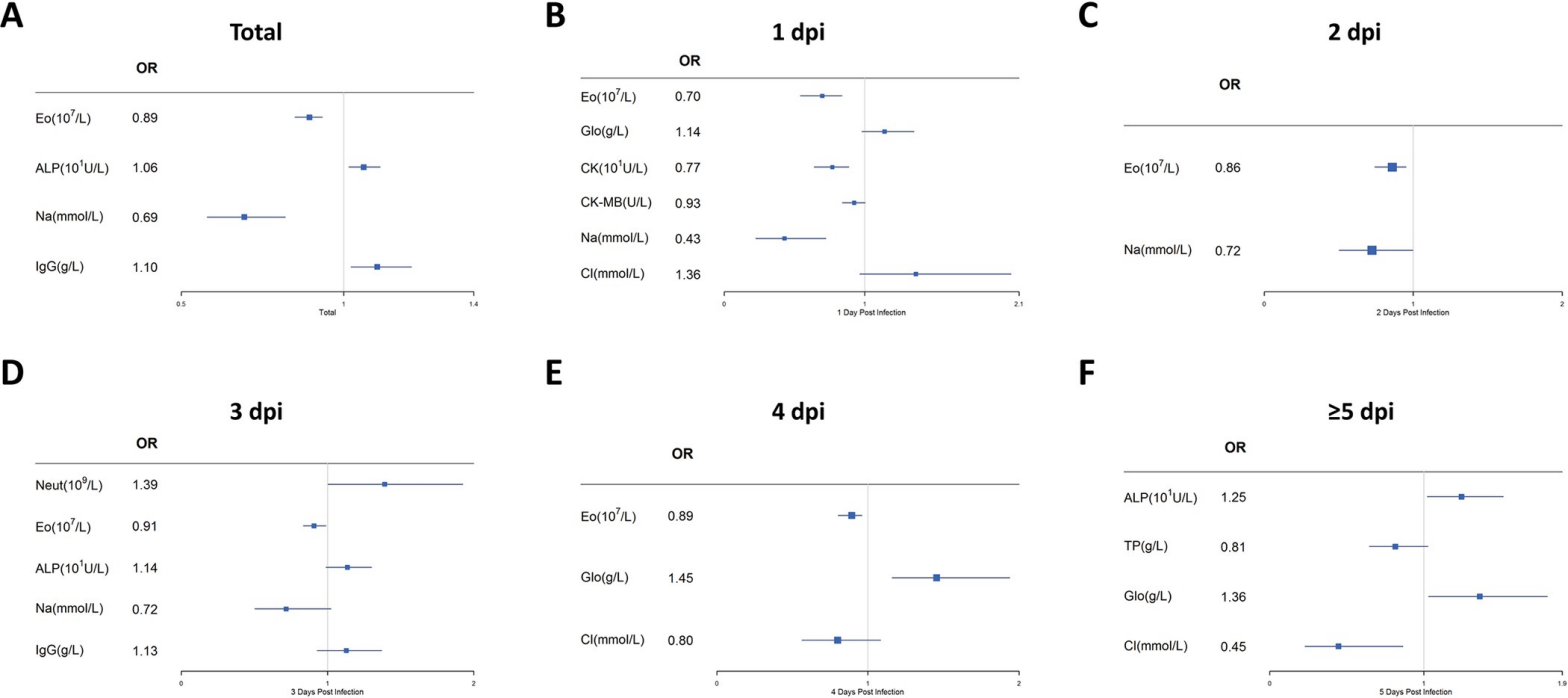
Multivariable logistic regression analysis of predictors for severe HFMD. Blood test results were analyzed by logistic regression to assess the potential predictors for severe HFMD. (A). Logistic regression analysis of laboratory indicators from all mild (n = 178) and severe cases (n = 228). (B-E). We assessed predictors of severe HFMD by logistic regression model at 1 (B), 2 (C), 3 (D), 4 (E), and ≥5 dpi (F). **Abbreviations:** NEUT: neutrophils, EO: eosinophils, ALP: alkaline phosphatase, TP: total protein, GLO: globulin, CK: creatine kinase, CK-MB: creatine kinase isoenzyme, IgG: Immunoglobulin G.

We further analyzed above blood test results by logistic regression model to assess the potential predictors for severe HFMD. As shown in **Fig 4A**, for all mild and severe cases, the increased number of eosinophils (OR = 0.89, 95% CI [0.85~0.93], *P*<0.001) and the increased level of Na^+^ (OR = 0.69, 95% CI [0.58~0.82], *P*<0.001) were independent protective factors for severe HFMD, while the rising levels of alkaline phosphatase (ALP) (OR = 1.06, 95% CI [1.01~1.11], *P* = 0.01) and IgG (OR = 1.10, 95% CI [1.02~1.20], *P* = 0.02) were risk factors for severe HFMD. To define the role of immune system and metabolism, and inflammatory biomarkers in HFMD development, we further assessed those laboratory findings by multivariable logistic regression model at different onset time. The increased number of eosinophils was still an independent protective factor for severe HFMD at 1 (OR = 0.69, 95% CI [0.54~0.89], *P*<0.001) (**Fig 4B**), 2(OR = 0.85, 95% CI [0.80~0.96], *P* = 0.02) (**Fig 4C**), 3 (OR = 0.91, 95% CI [0.83~0.99], *P* = 0.03) (**Fig 4D**) and 4 dpi (OR = 0.89, 95% CI [0.85~0.93], *P* = 0.01) (**Fig 4E**). In addition, the increased levels of Na^+^ (OR = 0.43, 95% CI [0.23~0.72], *P* = 0.004) (**Fig 4B**), Cl^-^(OR = 0.45, 95% CI [0.23~0.87], *P* = 0.02) (**Fig 4B**) and CK (OR = 0.77, 95% CI [0.64~0.89], *P* = 0.001)(**Fig 4F)** were protective factors at 1 and ≥5 dpi. The elevated level of globulin was a risk factor of severe HFMD at 4 dpi (OR = 1.45, 95% CI [1.16~1.94], *P* = 0.004) (**Fig 4E)** and ≥5 dpi (OR = 1.36, 95% CI [1.03~1.80], *P* = 0.03) (**Fig 4F)**. The increased number of neutrophils (OR = 1.39, 95% CI [1.004~1.93], *P* = 0.047) (**Fig 4D)** and the level of alkaline phosphatase (ALP) (OR = 1.25, 95% CI [1.02~1.52], *P* = 0.03) (**Fig 4F)** were signs of risk for severe HFMD at 3 and ≥5 dpi, respectively. Together, our results suggest that reduced number of eosinophils, decrease in Na^+^, Cl^-^, and creatine kinase concentrations, increased number of neutrophils and concentrationof ALP, IgG and globulin are predictors for severe HFMD.

## Discussion

Outbreak of HFMD is a threat to the health of infants and young children worldwide [1, 2, 5, 16, 17]. Fatal cases always come from infants and young children with severe symptoms, such as brainstem encephalitis, aseptic meningitis, encephalitis, acute flaccid paralysis (AFP), and acute cardiopulmonary failure [6, 18]. Discovering early relevant clinical and/or laboratory test signs that associate with severe symptoms will be very beneficial to design a treatment plan for early medical intervention to reduce mortality. In this study, we found that rural living, hyperpyrexia, immune changes including reduced number of eosinophils, increased number of neutrophils and levels of IgG and globulin, metabolic alterations including increased level of ALP, loss of blood Na^+^ and Cl^-^, and decreased level of creatine kinase in the peripheral blood were predictors of severe HFMD.

Firstly, this study revealed that cases from rural area and with hyperpyrexia seemed more susceptible to severe symptoms, which was consistent with a previous study [7]. Early diagnosis and treatment should be beneficial for those cases from rural area. Changes in immune cells, including neutrophils, macrophages, T cells and dendritic cells, have been reported to be associated with HFMD. These observations were supported by the evidence from *in vitro* and *in vivo* studies [19–21]. In this study, we found that the increased number of neutrophils was linked to severe HFMD at 3 dpi. During the early phase of inflammatory response, particularly during bacterial or viral infection, neutrophils are on the first line of defense against foreign invaders and are recruited to the site of inflammation by proinflammatory cytokines or chemokines [22]. Eosinophils make up a small proportion of white blood cells. It is thought that these cells mostly function in host defense against parasites and during allergic responses; however, they are also involved in fighting viral infections [23]. Our results indicate that the number of eosinophils was significantly reduced in patients with the risk of severe HFMD. The reason for this is not clear. It is possible that these eosinophils migrate from blood to infection sites or that overproduction of inflammatory cytokines may affect eosinophil differentiation or survival. IgG, produced by plasma B cells, is the main component of antibodies found in blood and extracellular fluid and plays important roles in host defense against pathogens. IgG can protect the body from infection by binding and neutralizing the invaded pathogens (e.g. viruses, bacteria, fungi) [24]. Our data suggestthat the increased level of IgG and other immunoglobulins were predictors for severe HFMD at 3 dpi. The high level of IgG indicate a strong B cell response, which may be required to clear serious viral infection in the patients with severe HFMD.

During the pathogenesis of HFMD, a wide variety of molecular and metabolic alterations have been recognized as an important event [10]. Our data indicate that electrolyte disorder may contribute to severe symptoms. Na^+^ and Cl^-^ are two major cation and anion inextracellular fluid. It is important that the concentration of sodiumchloride (NaCl) is maintained properly in extracellular fluid to balance the osmotic pressure between intracellular and extracellular environments. Hyponatremia happens when water loss exceeds NaCl loss [25]. In our study, we found that loss of blood Na^+^ and Cl^-^ was associated with the risk of severe HFMD, which confirmed hyponatremia in severe cases. Hyponatremia can lead to increase in osmosis and tissue cells swell (edema), and may also further cause heart failure [26]. It has been reported that young children with neural infection are prone to hyponatremia [27]. These data suggest that hyponatremia may be associated with neural lesions and fatal cardiopulmonary failure in severe cases. Thus, management of fluids is critical to prevent or limit severe cases. Alkaline phosphatase is an enzyme that functions to transport metabolites across cell membranes. Liver and bone diseases are the most common causes of pathological elevation of ALP, although itmay also be released by other tissues [28]. In our study, we found that the elevated level of ALP was a predictor of severe HFMD, which was in agreement with previous studies [29, 30]. We speculate that mild liver injury-induced by viral infection or drug intake during hospitalization might be responsible for increased ALP in severe cases. High concentration ofcreatine kinase(CK) in the blood is an indicator of damage to CK-rich tissue, such as in rhabdomyolysis, myocardial infarction, myositis and myocarditis [31]. However, in cases of severe HFMD, the level of this enzyme was actually reduced. Why these patients had reduced creatine kinase in their blood is not clear.

## Conclusion

In summary, our study suggests that rural living, hyperpyrexia, immune changes including lower number of eosinophils, increased number of neutrophils and levels of IgG and globulin, metabolic alterations including increased level of alkaline phosphatase, loss of blood Na^+^ and Cl^-^, and decreased level of creatine kinase in peripheral blood are predictors for severe HFMD. Fluid should be managed as early as possible to protect severe cases developing into fatal cardiopulmonary edema. Of course, our study also has some shortcomings. Patients treated with different medicines can affect our results. In the future, more prospective studies should be conducted to understand the underlying risk factors for severe HFMD.

## Supporting information

S1 File STROBE checklist. (DOCX)
