# Supplementary material for: Identification of immune and metabolic predictors of severe hand-foot-mouth disease
Source: PLoS One. 2019 May 23;14(5):e0216993. doi: 10.1371/journal.pone.0216993 (PMC6532886; doi:10.1371/journal.pone.0216993)
Supplement: S1 File — (DOCX) [file pone.0216993.s001.docx]

STROBE Statement—checklist of items that should be included in reports of observational studies

|  | | Item No. | | Recommendation | Page  No. | | Relevant text from manuscript |
| --- | --- | --- | --- | --- | --- | --- | --- |
| **Title and abstract** | | 1 | | (*a*) Indicate the study’s design with a commonly used term in the title or the abstract | 1 | | a case-control study |
|  |  |  |  | (*b*) Provide in the abstract an informative and balanced summary of what was done and what was found | 1 | | 406 mild and severe patients were recruited and divided into different subgroups based on the number of days from the initial onset time to hospitalization (1, 2, 3, 4, and ≥5 days ). Logistic regression was used to define the risk factors of severe HFMD. Our results suggested that rural living, hyperpyrexia, changes in the immune system that include the numbers of eosinophils and neutrophils and the levels of IgG and globulin, and metabolic alterations, such as the levels of alkaline phosphatase, Na^+^, Cl^-^, and creatine kinase in peripheral blood are predictors of severe HFMD. |
| Introduction | | | | | | |  |
| Background/rationale | | 2 | | Explain the scientific background and rationale for the investigation being reported | 2 | | Immune and metabolic changes have been detected in HFMD patients However, the association of these changes with HFMD severity has not been demonstrated. Although the inactivated EV71 vaccine have been widely inoculated in infants and young children in several provinces of mainland China, which exhibits effective protection against EV71-associated severe HFMD, the number of severe cases is still large, posing a threat to early infancy and young children in rural area of Asia-Pacific region |
| Objectives | | 3 | | State specific objectives, including any prespecified hypotheses | 3 | | Understanding which immune and metabolic alterations contribute to the risk of severe HFMD will help to better inform public health and clinical practice. |
| Methods | | | | | | |  |
| Study design | | 4 | | Present key elements of study design early in the paper | 3, 4 | | A total of 406 subjects including 178 mild cases and 228 severe cases was enrolled in this study. These patients were admitted in the Children's Hospital of Zhengzhou from January 2015 to December 2016. HFMD cases were diagnosed following “hand, foot and mouth disease treatment guidelines” (Chinese Ministry of Public Health, revised in 2010). The cases with encephalitis, acute flaccid paralysis, myocarditis, and pulmonary edema were classified as severe cases, and the cases without above symptoms were classified as mild cases. Additionally, the patients with congenital heart disease, pneumonia, and autoimmune disease were excluded from this study. |
| Setting | | 5 | | Describe the setting, locations, and relevant dates, including periods of recruitment, exposure, follow-up, and data collection | 3 | | A total of 406 subjects including 178 mild cases, 228 severe cases were enrolled in this study. These patients were admitted in the Children's Hospital of Zhengzhou from January 2015 to December 2016.  Demographic characteristics, clinical symptoms were collected by the physicians through retrospective medical records and face-to-face interviews with children’s parents during the admission. The clinical information of HFMD cases was collected after onset of illness, but before diagnosis of severe HFMD or fatality. |
| Participants | | 6 | | (*a*) *Cohort study*—Give the eligibility criteria, and the sources and methods of selection of participants. Describe methods of follow-up  *Case-control study*—Give the eligibility criteria, and the sources and methods of case ascertainment and control selection. Give the rationale for the choice of cases and controls  *Cross-sectional study*—Give the eligibility criteria, and the sources and methods of selection of participants | 3 | | HFMD cases were diagnosed following “hand, foot and mouth disease treatment guidelines” (Chinese Ministry of Public Health, revised in 2010). The cases with encephalitis, acute flaccid paralysis, myocarditis and pulmonary edema were classified as severe cases (Cases group). The cases without severe symptoms are as controls. Additionally, the cases with congenital heart disease, pneumonia and autoimmune diseases were excluded. |
|  |  |  |  | (*b*) *Cohort study*—For matched studies, give matching criteria and number of exposed and unexposed  *Case-control study*—For matched studies, give matching criteria and the number of controls per case |  | |  |
| Variables | | 7 | | Clearly define all outcomes, exposures, predictors, potential confounders, and effect modifiers. Give diagnostic criteria, if applicable | 3 | | HFMD cases were diagnosed following “hand, foot and mouth disease treatment guidelines” (Chinese Ministry of Public Health, revised in 2010). Recalling bias, reporting bias, diagnostic suspicion bias and detection bias should be main confounders. |
| Data sources/ measurement | | 8* | | For each variable of interest, give sources of data and details of methods of assessment (measurement). Describe comparability of assessment methods if there is more than one group | 4 | | Laboratory indicators were collected from general blood tests for clinical diagnosis. Difference between groups or subgroups was tested by Student’s t test or Kruskal-Wallis test according to the distribution of data. Chisquare test or Fisher’s exact test was used to test the differences in the proportions of categorical variables. |
| Bias | | 9 | | Describe any efforts to address potential sources of bias |  | | Researchers should develop detailed data collection methods and strict quality control methods for the proposed research. Collect data using the “blind method”. Strictly investigate the scientific attitude of designers and researchers |
| Study size | | 10 | | Explain how the study size was arrived at |  | | According to our previous work N=Z 2 ×(P ×(1-P))/E 2 |
| Quantitative variables | 11 | | Explain how quantitative variables were handled in the analyses. If applicable, describe which groupings were chosen and why | | 6 | Student’s *t* test or Kruskal-Wallis test | |
| Statistical methods | 12 | | (*a*) Describe all statistical methods, including those used to control for confounding | | 6 | Student’s *t* test, Kruskal-Wallis, Chisquare test and Fisher’s exact test | |
|  |  |  | (*b*) Describe any methods used to examine subgroups and interactions | | 6 | Student’s *t* test, Kruskal-Wallis | |
|  |  |  | (*c*) Explain how missing data were addressed | |  | No application | |
|  |  |  | (*d*) *Cohort study*—If applicable, explain how loss to follow-up was addressed  *Case-control study*—If applicable, explain how matching of cases and controls was addressed  *Cross-sectional study*—If applicable, describe analytical methods taking account of sampling strategy | |  | No application | |
|  |  |  | (*e*) Describe any sensitivity analyses | |  | No application | |
| Results | | | | | | | |
| Participants | 13* | | (a) Report numbers of individuals at each stage of study—eg numbers potentially eligible, examined for eligibility, confirmed eligible, included in the study, completing follow-up, and analysed | |  | No application | |
|  |  |  | (b) Give reasons for non-participation at each stage | |  | No application | |
|  |  |  | (c) Consider use of a flow diagram | |  | No application | |
| Descriptive data | 14* | | (a) Give characteristics of study participants (eg demographic, clinical, social) and information on exposures and potential confounders | | 3 | A total of 406 subjects including 178 mild cases, 228 severe cases were enrolled in this study. These patients were admitted in the Children's Hospital of Zhengzhou from January 2015 to December 2016. HFMD cases were diagnosed following “hand, foot and mouth disease treatment guidelines” (Chinese Ministry of Public Health, revised in 2010) | |
|  |  |  | (b) Indicate number of participants with missing data for each variable of interest | |  | No application | |
|  |  |  | (c) *Cohort study*—Summarise follow-up time (eg, average and total amount) | |  | No application | |
| Outcome data | 15* | | *Cohort study*—Report numbers of outcome events or summary measures over time | |  | No application | |
|  |  |  | *Case-control study—*Report numbers in each exposure category, or summary measures of exposure | |  | No application | |
|  |  |  | *Cross-sectional study—*Report numbers of outcome events or summary measures | |  | No application | |
| Main results | 16 | | (*a*) Give unadjusted estimates and, if applicable, confounder-adjusted estimates and their precision (eg, 95% confidence interval). Make clear which confounders were adjusted for and why they were included | | 7, 13 | Rural living (OR=1.76, 95% CI [1.19~2.63], *P*=0.005); >39°C body temperature (OR=2.14, 95% CI [1.12~4.12], *P*=0.022); eosinophils (OR=0.89, 95% CI [0.85~0.93], *P*<0.001); Na (OR=0.69, 95% CI [0.58~0.82], *P*<0.001); ALP (OR=1.06, 95% CI [1.01~1.11], *P*=0.01) and IgG (OR=1.10, 95% CI [1.02~1.20], *P*=0.02) | |
|  |  |  | (*b*) Report category boundaries when continuous variables were categorized | |  | No application | |
|  |  |  | (*c*) If relevant, consider translating estimates of relative risk into absolute risk for a meaningful time period | |  | No application | |

Continued on next page

| Other analyses | 17 | Report other analyses done—eg analyses of subgroups and interactions, and sensitivity analyses | 10-13 | Compared to mild cases, the numbers of basophils (at 2, 4 dpi, Fig 1D) and eosinophils (at 1, 2, 3, 4 dpi, Fig 1E) in peripheral blood of severe cases were all significantly reduced, while the number of neutrophils in peripheral blood of severe cases was significantly increased at 3 dpi. In addition, in response to serious viral infection, the levels of globulin (at 1, 3, 4, ≥5 dpi, Fig 1F) and IgG (at 3 dpi, Fig 1H) in peripheral blood of severe cases were all significantly elevated, compared to mild cases. In comparison with mild cases, the level of ALP in peripheral blood of severe cases was significantly increased at 3 and 5 dpi, while the levels of CK (at 1 dpi), CK-MB (at 1 dpi), Na (at 1, 2, 3, ≥5 dpi), Cl (at 1, 4, ≥5 dpi) in peripheral blood of severe cases was significantly reduced. The level of total protein (at 1, 3, 4, ≥5 dpi) in peripheral blood of severe cases was significantly elevated, compared to mild cases. The increased number of eosinophils was still independent protect factors for severe HFMD at 1 (OR=0.69, 95% CI [0.54~0.89], *P*<0.001), 2(OR=0.85, 95% CI [0.80~0.96], *P*=0.02), 3 (OR=0.91, 95% CI [0.83~0.99], *P*=0.03) and 4 dpi (OR=0.89, 95% CI [0.85~0.93], *P*=0.01). In addition, the increased levels of Na (OR=0.43, 95% CI [0.23~0.72], *P*=0.004), Cl (OR=0.45, 95% CI [0.23~0.87], *P*=0.02) and CK (OR=0.77, 95% CI [0.64~0.89], *P*=0.001) were protect factors at 1 and ≥5 dpi. The elevated level of globulin was risk factor of severe HFMD at 4 dpi (OR=1.45, 95% CI [1.16~1.94], *P*=0.004) and ≥5 dpi (OR=1.36, 95% CI [1.03~1.80], *P*=0.03). The increased neutrophils number (OR=1.39, 95% CI [1.004~1.93], *P*=0.047) and ALP level (OR=1.25, 95% CI [1.02~1.52], *P*=0.03) posed risk of severe HFMD at 3 and ≥5 dpi, respectively. |
| --- | --- | --- | --- | --- |
| Discussion | | | | |
| Key results | 18 | Summarise key results with reference to study objectives | 14 | In summary, our study suggests that rural living, hyperpyrexia, immune changes including lower number of eosinophils, increased number of neutrophils and levels of IgG and globulin, metabolic alterations including increased level of alkaline phosphatase, loss of blood Na^+^ and Cl^-^, and decreased level of creatine kinase in peripheral blood are predictors for severe HFMD. |
| Limitations | 19 | Discuss limitations of the study, taking into account sources of potential bias or imprecision. Discuss both direction and magnitude of any potential bias | 16 | Patients with different drug intake or treatment can also influence our results. |
| Interpretation | 20 | Give a cautious overall interpretation of results considering objectives, limitations, multiplicity of analyses, results from similar studies, and other relevant evidence | 15, 16 | Firstly, this study revealed that cases from rural area and with hyperpyrexia seemed more susceptible to severe symptoms, which was consistent with a previous study. Early diagnosis and treatment should be beneficial for those cases from rural area. Changes in immune cells, including neutrophils, macrophages, T cells and dendritic cells, have been reported to be associated with HFMD. These observations were supported by the evidence from in vitro and in vivo studies. In this study, we found that the increased number of neutrophils was linked to severe HFMD at 3 dpi. During the early phase of inflammatory response, particularly during bacterial or viral infection, neutrophils are on the first line of defense against foreign invaders and arerecruited to the site of inflammation by proinflammatory cytokines or chemokines. Eosinophils make up a small proportion of white blood cells. It is thought that these cells mostly function in host defense against parasites and during allergic responses; however, they are also involved in fighting viral infections. Our results indicate that the number of eosinophils was significantly reduced in patients with the risk of severe HFMD. The reason for this is not clear. It is possible that these eosinophils migrate from blood to infection sites or that overproduction of inflammatory cytokines may affect eosinophil differentiation or survival. IgG, produced by plasma B cells, is the main component of antibodies found in blood and extracellular fluid and plays important roles in host defense against pathogens. IgG can protect the body from infection by binding and neutralizing the invaded pathogens (e.g. viruses, bacteria, fungi). Our data suggest that the increased level of IgG and other immunoglobulins werepredictors for severe HFMD at 3 dpi. The high level of IgG indicate a strong B cell response, which may be required to clear serious viral infection in the patients with severe HFMD.  During the pathogenesis of HFMD, a wide variety of molecular and metabolic alterations have been recognized as an important event. Our data indicate that electrolyte disorder may contribute to severe symptoms. Na+ and Cl- are two major cation and anion in extracellular fluid. It is important that the concentration of sodium chloride (NaCl) is maintained properly in extracellular fluid to balance the osmotic pressure between intracellular and extracellular environments. Hyponatremia happens when water loss exceeds NaCl loss. In our study, we found that loss of blood Na+ and Cl- was associated with the risk of severe HFMD, which confirmed hyponatremia in severe cases. Hyponatremia can lead to increase in osmosis and tissue cells swell (edema), and may also further cause heart failure [26]. It has been reported that young children with neural infection are prone to hyponatremia. These data suggest that hyponatremia may be associated with neural lesions and fatal cardiopulmonary failure in severe cases. Thus, management of fluids is critical to prevent or limit severe cases. Alkaline phosphatase is an enzyme that functions to transport metabolites across cell membranes. Liver and bone diseases are the most common causes of pathological elevation of ALP, although it may also be released by other tissues. In our study, we found that the elevated level of ALP was a predictor of severe HFMD, which was in agreement with previous studies. We speculate that mild liver injury-induced by viral infection or drug intake during hospitalization might be responsible for increased ALP in severe cases. High concentration of creatine kinase (CK) in the blood is an indicator of damage to CK-rich tissue, such as in rhabdomyolysis, myocardial infarction, myositis and myocarditis. However, in cases of severe HFMD, the level of this enzyme was actually reduced. Why these patients had reduced creatine kinase in their blood is not clear. |
| Generalisability | 21 | Discuss the generalisability (external validity) of the study results |  | No application |
| Other information | |  | | |
| Funding | 22 | Give the source of funding and the role of the funders for the present study and, if applicable, for the original study on which the present article is based | 16 | This work was funded by the National Natural Science Foundation of China (81172740, Guangcai Duan); National Natural Science Foundation of China (81573205, Guangcai Duan); Key Scientific Research Projects in Colleges and Universities of Henan Province (15A330003, Jingchao Ren); Outstanding Doctoral Thesis Training Fund of Zhengzhou University (201511270165, Yuefei Jin). |

*Give information separately for cases and controls in case-control studies and, if applicable, for exposed and unexposed groups in cohort and cross-sectional studies.

**Note:** An Explanation and Elaboration article discusses each checklist item and gives methodological background and published examples of transparent reporting. The STROBE checklist is best used in conjunction with this article (freely available on the Web sites of PLoS Medicine at http://www.plosmedicine.org/, Annals of Internal Medicine at http://www.annals.org/, and Epidemiology at http://www.epidem.com/). Information on the STROBE Initiative is available at www.strobe-statement.org.
